# The Role of microRNAs in the *Drosophila Melanogaster* Visual System

**DOI:** 10.3389/fcell.2022.889677

**Published:** 2022-04-04

**Authors:** Davide Colaianni, Cristiano De Pittà

**Affiliations:** Department of Biology, University of Padova, Padova, Italy

**Keywords:** MicroRNAs, *Drosophila melanogaster*, visual system, optic lobe, compound eye, circadian rhythms

## Abstract

MicroRNAs (miRNAs) are a class of small non-coding RNAs (∼22 nucleotides in length) that negatively regulate protein-coding gene expression post-transcriptionally by targeting mRNAs and triggering either translational repression or RNA degradation. MiRNA genes represent approximately 1% of the genome of different species and it has been estimated that every miRNA can interact with an average of 200 mRNA transcripts, with peaks of 1,500 mRNA targets per miRNA molecule. As a result, miRNAs potentially play a fundamental role in several biological processes including development, metabolism, proliferation, and apoptotic cell death, both in physiological and pathological conditions. Since miRNAs were discovered, *Drosophila melanogaster* has been used as a model organism to shed light on their functions and their molecular mechanisms in the regulation of many biological and behavioral processes. In this review we focus on the roles of miRNAs in the fruit fly brain, at the level of the visual system that is composed by the compound eyes, each containing ∼800 independent unit eyes called ommatidia, and each ommatidium is composed of eight photoreceptor neurons that project into the optic lobes. We describe the roles of a set of miRNAs in the development and in the proper function of the optic lobes (*bantam*, *miR-7*, *miR-8*, *miR-210*) and of the compound eyes (*bantam*, *miR-7*, *miR-9a*, *miR-210*, *miR-263a/b*, *miR-279/996*), summarizing also the pleiotropic effects that some miRNAs exert on circadian behavior.

## Introduction

### microRNAs Biogenesis and Function in *Drosophila Melanogaster*

MicroRNAs (miRNAs) are a class of small non-coding RNAs (∼22 nucleotides in length) that negatively regulate protein-coding gene expression post-transcriptionally by targeting mRNAs, mostly at the 3′ untranslated region (3′-UTR) and triggering either translational repression or RNA degradation. MiRNA genes represent approximately 1% of the genome of different species and every microRNA has hundreds of targets playing a fundamental role in several biological processes including development, metabolism, proliferation, and apoptotic cell death (He and Hannon, 2004). In the canonical pathway, most miRNA genes are transcribed in the nucleus by RNA polymerase II; the resulting primary miRNA (pri-miRNA) is processed by Drosha and its partner Pasha, which together form the microprocessor complex that is responsible for the cleavage of the pri-miRNA into a 60–70 nucleotide hairpin structure known as precursor miRNA (pre-miRNA) (Wahid et al., 2010). In addition, pre-miRNA-like hairpins can also be generated by the mirtron pathway (Okamura et al., 2007), merging with the canonical pathway when the pre-miRNA is exported into the cytoplasm through EXP5. In the cytoplasm, the pre-miRNA is further processed by Dcr-1 that together with Loqs and AGO1, is responsible for the cleavage of the pre-miRNA hairpin loop and the formation of a miRNA/miRNA* duplex. After cleavage, the passenger strand (miRNA*) is usually degraded, while the guide strand, the mature form of the miRNA, is loaded into AGO1, forming a ribonucleoprotein complex (RISC). Once assembled, the mature miRNA drives RISC toward its mRNA targets which show full or partial sequence complementarity with its seed region and are silenced through mRNA degradation or translation repression (Wahid et al., 2010).

### Fly Visual System Development and Anatomy

The fly visual system, composed by the compound eyes and the optic lobes, contains ∼150,000 neurons and glial cells, comprising more than 60% of the neurons of its central nervous system (Nériec and Desplan, 2016).

### The Compound Eye Development and Anatomy

**FIGURE 1:**
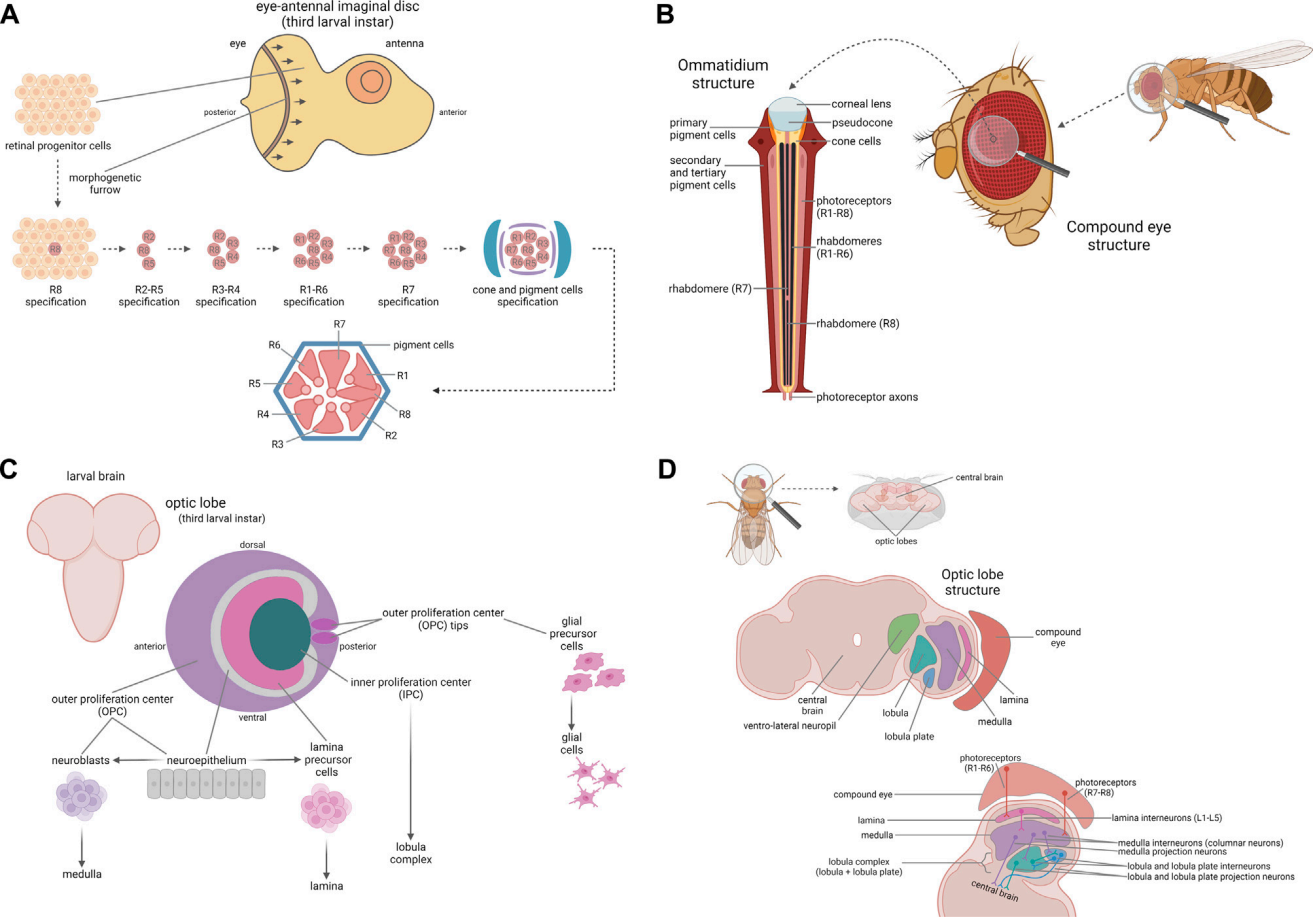
Fly visual system. Development and anatomy of the compound eye. **(A)** The compound eye is derived from the eye-antennal imaginal disc, an epithelium giving rise to both the eye and the antenna. During the third larval instar, a posterior-to-anterior wave of differentiation occurs in the eye portion, leading to the differentiation of the retinal progenitor cells into all the cell types, depending on their positions. The first cells undergoing specification are the photoreceptors, beginning with R8, followed by R2/R5, R3/R4, R1/R6, and R7, respectively, cone cells and pigment cells, which assemble to form the ommatidium final structure, here represented in cross-section. **(B)** The compound eye is composed of ∼800 units called ommatidia. Each ommatidium is composed of a corneal lens, a pseudocone, as well as cone and pigment cells, which form its backbone and are essential to focus the light on the photoreceptor neurons. In each ommatidium there are eight photoreceptor neurons (R1-R8), six “outer” (R1-R6) and two “inner” (R7-R8) ones, which are responsible for photoreception and for signal transduction from the retina to the optic lobes. Development and anatomy of the optic lobe. **(C)** The optic lobe is derived from a plate-like structure named optic placode and it is organized into the outer (OPC) and inner proliferation center (IPC). During larval development, the IPC and OPC give rise to the lobula complex and to the lamina, the medulla, and the glial cells respectively. The neuroepithelial cells composing the neuroepithelium of the OPC are converted into the lamina precursor cells in the inner part of the OPC and into the neuroblasts which in turn generate the neurons forming the medulla in the medial part of the OPC. In addition, the tips of the OPC represent the glial precursor cell areas, which will differentiate into the glial cells of the optic lobe. **(D)** The optic lobe is composed of four neuropils: the lamina, the medulla, the lobula and the lobula plate. The lamina is localized near the retina and is innervated by the outer photoreceptors R1-R6; lamina interneurons L1-L5, targeted by R1-R6 axons, project to the medulla. The medulla is innervated by the inner photoreceptors R7 and R8 and by the lamina interneurons L1-L5; the medulla, similarly to the lamina, is mainly composed of interneurons, and in particular of columnar neurons projecting into the lobula complex. The lobula and the lobula plate, on the other hand, are mainly composed of projection neurons, which transmit the visual information to the ventro-lateral neuropils in the central brain for the last step of visual processing. Created with BioRender.com.

The compound eye is derived from an eye-antennal imaginal disc, in which all cells undergo undifferentiated growth until the third larval instar, when a posterior-to-anterior wave of differentiation (morphogenetic furrow) occurs in the eye portion. The retinal progenitor cells start to differentiate because of their position, acquiring specific fates within the ommatidial rudiments by the action of the retinal determination gene network (RDGN) (Kumar, 2010). This developmental process leads to the definition of all the cell types, from the photoreceptor neurons (beginning with R8, followed by R2/R5, R3/R4, R1/R6, and R7 respectively), to the cone cells and the primary, secondary and tertiary pigment cells (Cagan, 2009) (Figure 1A).

The compound eye is composed of ∼800 units named ommatidia, arranged in an oval shape. Each ommatidium is composed of eight photoreceptor neurons (R1-R8), four cone cells, and two primary pigment cells. In addition, each ommatidium shares six secondary pigment cells and three tertiary pigment cells (also named interommatidial cells) with its surrounding neighbors (Figure 1B). Cone and primary pigment cells are two subsets of non-neuronal cells that secrete the corneal lens and the underlying pseudocone, an extracellular fluid-filled cavity which acts, along with secondary and tertiary pigment cells, to limit light scattering and to focus the light on photoreceptor neurons (Charlton-Perkins and Cook, 2010; Kumar, 2012) that are the specialized neuroepithelial cells responsible for visual phototransduction (Montell, 2012). Among the eight photoreceptors, R1-R6 and R7-R8 are localized in the outer regions and in the central region respectively. Since R7 is localized above R8, only seven photoreceptors are visible in the ommatidium cross-section. The photoreceptor nucleus and organelles reside in the cell body, while photoreception and signal transduction take place in the rhabdomere, a specialized compartment organized in a closely packed stack of ∼50,000 microvilli (Kumar, 2012). Finally, photoreceptor axons project from the retina to the optic lobes for visual information processing (Chang et al., 2018).

### The Optic Lobe Development and Anatomy

The optic lobe precursor cells, derived from the neurectoderm of the embryonic head, form a plate-like structure named optic placode, which is then separated into two layers, the outer (OPC) and the inner proliferation center (IPC). After a long period of proliferation until the early pupal stage, the neuroepithelium of the medial part of the OPC is converted into the neuroblasts that generate the neurons forming the medulla, the inner part of the OPC generates the neurons forming the lamina, and the IPC generates the neurons forming the lobula complex (Figure 1C). So, 40 h after puparium formation, the optic lobe reaches its final structure, composed of the four neuropils: lamina, medulla, lobula and lobula plate. The lamina is localized near the retina and is innervated by R1-R6 photoreceptors, responsible for motion detection. Lamina interneurons L1-L5, targeted by R1-R6 photoreceptors axons, project to the medulla. The medulla is organized into distal and proximal medulla and the first one is innervated by the inner photoreceptors R7 and R8, responsible for color vision, and by the lamina interneurons L1-L5, while the proximal medulla receives information from the distal medulla and further computes visual information. Among all the medulla interneurons, only columnar neurons exit from the medulla, projecting into the lobula complex. The lobula complex comprise both the lobula and the lobula plate. In contrast with lamina and medulla, the lobula and the lobula plate are mostly composed of projection neurons, which connect the optic lobe to the central brain. In the lobula plate, involved in motion visual processing, some interneurons (T4 and T5) form connections between lobula plate, lobula and medulla, while the projection neurons transmit visual information to the ventro-lateral neuropils in the central brain, for the last step of visual processing (Nériec and Desplan, 2016; Ngo et al., 2017) (Figure 1D).

Interestingly, some miRNAs play fundamental roles in the development and homeostasis of the fly visual system and also exert a role on its circadian behavior, which is closely related to the capacity of the fly to perceive light and to synchronize to day-night cycles (Rieger et al., 2003).

### *Drosophila Melanogaster* Circadian Clock

Circadian rhythms are a set of physiological and behavioral changes following a cycle of ∼24 h, occurring in most organisms from cyanobacteria to humans and have been extensively studied in *D. melanogaster*, since the circadian clock influences many of the fruit fly’s behaviors (e.g., locomotor activity). At the molecular level, circadian rhythms are generated by a negative transcriptional feedback loop: the circadian transcription factors CLOCK and CYCLE form a heterodimer that promotes the expression of its own repressors, PERIOD and TIMELESS, which go through various modifications until they are degraded to release CLK/CYC thus starting a new cycle. The stability of these molecules is regulated by a series of post-translational mechanisms, influencing the expression of other clock genes and of hundreds of clock-controlled genes responsible for the rhythmic physiological and behavioral outputs (Tataroglu and Emery, 2015). At the cellular level, the master clock is located in ∼150 neurons, which can be grouped into three major clusters: dorsal neurons (DN1-3), dorsal lateral neurons (LNds), small (s-LNvs) and large ventral lateral neurons (l-LNvs). Notably, a direct communication between the visual system and the circadian clock has been reported, with several photoreceptors targeting different subsets of clock neurons (Schlichting, 2020).

### The Role of microRNAs in the *Drosophila Melanogaster* Visual System

### *Bantam* (*ban*)

**FIGURE 2:**
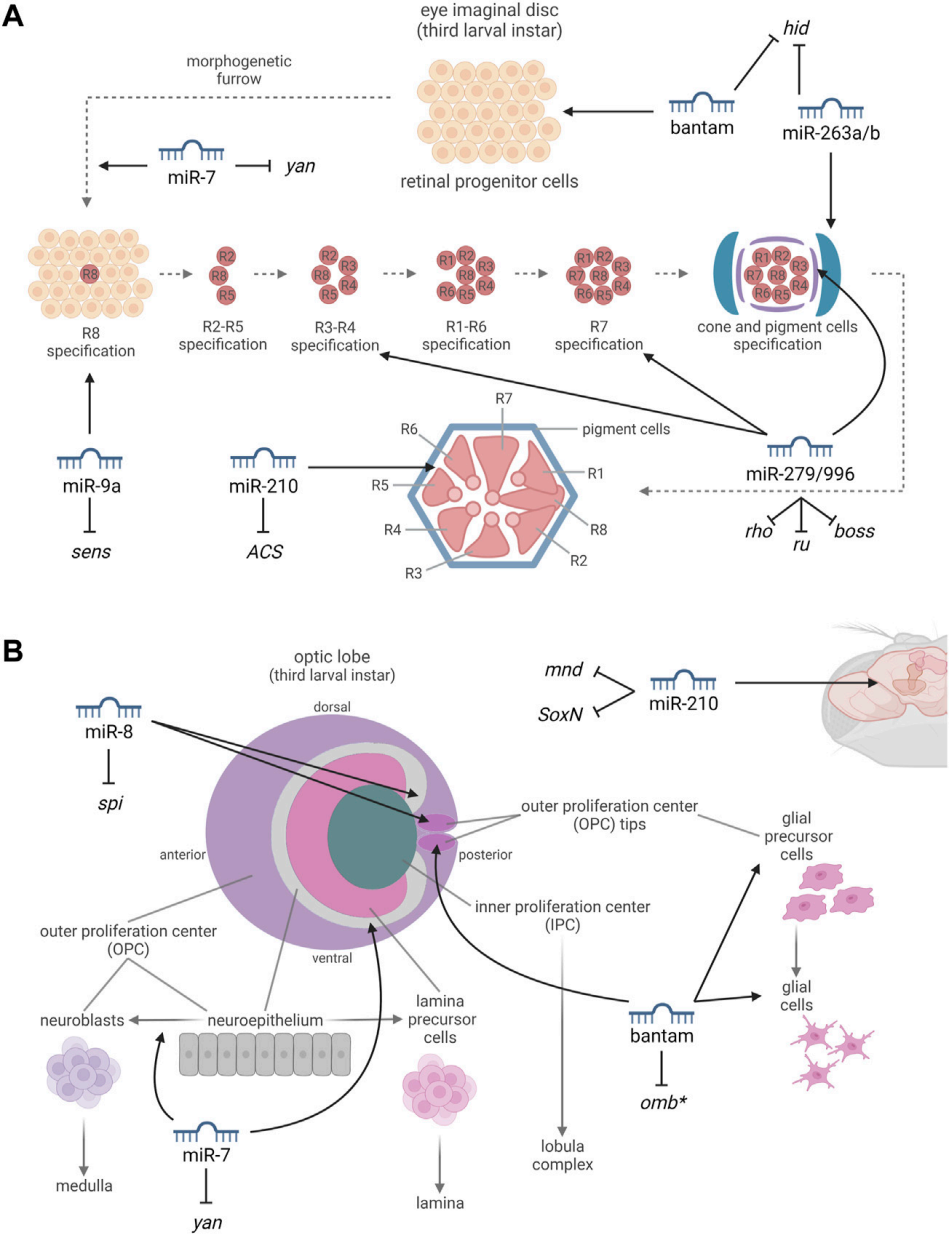
miRNAs involved in the development and in the proper functioning of the compound eye (A) and optic lobe (B). **(A)** By targeting the proapoptotic gene *hid*, bantam promotes cell proliferation and survival in the progenitor cells of the eye imaginal disc. miR-7, targeting *yan*, promotes a photoreceptor cell fate. miR-9a, targeting *sens*, is thought to be involved in the differentiation of the R8 photoreceptor, which occurs by a neuronal selection process such as is observed in the sensory organ precursor cells (SOPs). miR-279/996, targeting *rho*, *ru*, and *boss*, are required for the definition of ommatidial cell fates. miR-263a/b, targeting *hid*, protect the developing sense organs and interommatidial cells (IOCs), from apoptosis. miR-210, targeting *ACS*, is essential to prevent progressive retinal degeneration. **(B)** miR-7, targeting *yan*, buffers the transition from neuroepithelial cells to neuroblasts. miR-8, expressed in the optic-lobe-associated cortex glia and targeting the growth factor *spi*, promotes the increase of glial size and is required to limit the growth and neuroblast formation within the neuroepithelium. By leading to the downregulation of *omb*, bantam is important for stem cell maintenance and for glial cell growth. miR-210, targeting *mnd* and *SoxN*, affects neurogenesis. *miRNA-mRNA interactions which have not been experimentally confirmed yet. Created with BioRender.com.

*Bantam* (*ban*) is part of several morphogen signaling pathways and specifically of the Hippo pathway, which controls tissue growth. *Ban* expression is activated by Yorkie (Yki) and is essential for Yki-induced overproliferation. In the uncommitted progenitor cells of the eye imaginal disc, Yki activates the expression of *ban* to promote cell proliferation and survival; on the other hand, *ban* expression is downregulated in cells undergoing either cell-cycle arrest or apoptosis, since it represses the translation of the proapoptotic gene *head involution defective* (*hid*) (Carthew et al., 2017; Peng et al., 2009) (Figure 2A). Consistently, over-expressing *ban* in the eye leads to an altered ocular phenotype characterized by bigger eyes (Bejarano and Lai, 2021). In addition, *ban* is highly expressed in the OPC of the developing optic lobes, where it is required for cell proliferation. In the glial precursor cell areas, *ban* is important for stem cell maintenance and for glial cell growth, increasing the proliferation of glial precursor and differentiated cells and affecting their distribution, largely through the down-regulation of the transcription factor *optomotor-blind* (*omb*) (Figure 2B). Consequently, *ban* expression affects the photoreceptor axon projection patterns to the optic lobes (Li and Padgett, 2012). Interestingly, *ban* has been also shown to be implicated in circadian rhythmicity, since it regulates the translation of *Clk* and *ban* over-expression induces circadian period lengthening (Li and Liu, 2014).

### miR-7

*miR-7* plays several different roles in flies, and it is involved in the regulation of the Hippo pathway inhibiting *yki* expression (Yu et al., 2020), establishing an interesting link with the previously discussed *ban* miRNA. In the eye, *miR-7* expression is activated at the beginning of photoreceptor differentiation as part of the EGFR pathway in a reciprocal negative feedback loop mechanism. In the undifferentiated retinal cells, when the transcription factor Yan is degraded, *miR-7* expression is activated and it in turn represses Yan activity by binding to its 3′-UTR. In this way, *Yan* is expressed in progenitor cells and *miR-7* is expressed in the developing photoreceptor cells, promoting photoreceptor cell fate at the expense of cone cell fate (Li and Carthew, 2005) (Figure 2A). In addition, in the developing optic lobe, the transition from neuroepithelial cells to neuroblasts, that in turn will generate the neurons forming the medulla, is buffered by *miR-7* which is expressed at the level of the neuroepithelial/neuroblast transition zone in response to EGF signaling (Figure 2B). *miR-7* limits Notch signaling through the repression of downstream Notch effectors including E(spl)m-γ, causing the switch from the proliferative division of the neuroepithelial cells to the differentiative division of the neuroblasts (Caygill and Brand, 2017).

### miR-8

*miR-8* is known to be a pleiotropic regulator of fruit fly ontogenesis, controlling physiological processes ranging from body size regulation to neuronal survival (Montigny et al., 2021). In the visual system, *miR-8* is expressed from the third larval instar at the level of the optic-lobe-associated cortex glia. It is required to limit the growth and neuroblast formation within the neuroepithelium through the modulation of the growth factor Spitz (*spi*) which triggers EGFR in order to promote neuroepithelial proliferation and neuroblast formation (Figure 2B). Thus, *miR-8* promotes the increase of glial size facilitating the sprouting toward the neuroepithelium and at the same time it negatively regulates *Spitz* (*spi*) preventing premature and excessive signaling in the underlying neuroepithelium (Morante et al., 2013).

### miR-9a

*miR-9a* has a key role in the commitment of the sensory organ precursor cells (SOPs) through the interaction with the 3′-UTR of *senseless* (*sens*). *Sens*, encodes for a zinc-finger transcription factor, and acts as a binary switch during SOPs selection, repressing or synergizing with proneural proteins on the basis of its expression levels (Li et al., 2006). In the ontogenesis of the fly eye, the neuronal selection leading to the differentiation of R8 photoreceptors is similar (although not identical) to that described for SOPs. In fact, *sens* is involved in the regulation of cell survival within the eye imaginal disc and is required for R8 photoreceptor differentiation (Gallicchio et al., 2021; Frankfort et al., 2001) (Figure 2A). Additionally, *miR-9a* over-expression in the eye results in an altered ocular phenotype characterized by heterogenous eye pigmentation (Bejarano and Lai, 2021), confirming its participation in the development of the fly visual system even if this has not been demonstrated yet.

### miR-210

In fruit flies, *miR-210* is preferentially expressed in the mushroom bodies, in the antennal lobes and in other olfactory organs, as well as in the main structures of the visual system, from the optic lobes to the compound eyes (Cusumano et al., 2018; Weigelt et al., 2019; Lyu et al., 2021). Weigelt et al. (2019) and Lyu et al. (2021) characterized the eye structure of *miR-210* knock-out (KO) flies and reported a progressive retinal degeneration, also accompanied by a reduction in photoreceptor function. In addition, transmission electron microscopy (TEM) analysis revealed the presence of abundant lipid droplet structures in the pigment cells of KO flies, which might represent the cause of the retinal degeneration and suggests the involvement of *miR-210* in lipid metabolism. Consistently, *Acetyl Coenzyme A synthase* (*ACS*), a homolog of *ACSS2*, involved in fat storage and utilization, was identified as a direct target of *miR-210* (Figure 2A). Nevertheless, additional unidentified targets are most likely yet to be discovered in order to explain the retinal degeneration. Interestingly, the overexpression of *miR-210* in clock cells during development led to an altered star shaped morphology of the l-LNvs cell bodies, aberrant arborisations in the optic lobe, and visual defects also affecting circadian rhythmicity. In this context, *minidiscs* (*mnd*), which encodes for an amino acid transporter required for imaginal disc growth (Martin et al., 2000), and *SoxNeuro* (*SoxN*), encoding a transcription factor that directs embryonic neural development (Ferrero et al., 2014), were identified as direct *miR-210* targets (Cusumano et al., 2018) (Figure 2B). Furthermore, *miR-210* over- and under-expression within clock neurons modulates circadian outputs such as the time of the morning and evening activity onset (Cusumano et al., 2018; Niu et al., 2019).

### *miR-263a* and *miR-263b*

*miR-263a/b* were found to protect the developing sense organs from apoptosis by down-regulating the proapoptotic gene *hid* and affecting apoptotic pruning, a process occurring to reduce the excess of unspecified progenitor cells during the development of compound eyes (Figure 2A). In this context, considering that 24 h after puparium formation around one out of three interommatidial cells (IOCs) undergo apoptosis, *miR-263a/b* expression ensure the survival of the remaining cells in order to guarantee the correct arrangement of the ommatidia (Hilgers et al., 2010). Consistently, *miR-263b* over-expressing flies showed bigger, more curved and very light colored eyes (Bejarano and Lai, 2021), confirming the effects of these miRNAs on IOCs which contain the pigment granules responsible for eye color (Tomlinson, 2012). Furthermore, both *miR-263a* and *miR-263b* are implicated in circadian rhythmicity, specifically in the regulation of locomotor activity (Nian et al., 2019; Nian et al., 2020).

### *miR-279* and *miR-996*

*miR-279* and *miR-996* have different sequences but they share similar seed regions and are involved in many functionally redundant cellular activities (Sun et al., 2015). *MiR-279/996* exert a prominent role in ommatidial cell fate determination in compound eyes. Indeed, *miR-279/996* ectopic expression results in the formation of degenerated R3/R4 photoreceptors with missing or vestigial rhabdomeres, while their deletion leads to the loss of cone cells and to the formation of ectopic R7 photoreceptors. It has been demonstrated that *miR-279/996* bind to the 3′-UTRs of multiple positive components of EGFR (*rhomboid* and *roughoid*) and Sevenless (*bride of sevenless*) signaling pathways impairing R7 photoreceptor specification (Duan et *al.*, 2018) (Figure 2A). In addition, *miR-279* and *miR-996* are implicated in the regulation of fruit fly rest/activity rhythms (Luo and Sehgal, 2012).

## Conclusion

The roles of a set of miRNAs involved in the development and proper functioning of the fruit fly visual system was explored. As regards the compound eye, *bantam* is expressed in the uncommitted progenitor cells of the eye imaginal disc where it promotes cell growth and survival until these cells start to differentiate (Carthew et al., 2017). The differentiation of the several cell types that compose the compound eye is mediated by different miRNAs: *miR-263a/b* ensure the survival of the proper number of IOCs (Hilgers et al., 2010), *miR-7* (Li and Carthew, 2005), *miR-9a* (Gallicchio et al., 2021), and *miR-279/996* (Duan et al., 2018) take part in photoreceptor differentiation, and *miR-210* was shown to be essential to photoreceptor maintenance (Lyu et al., 2021) (Figure 2A). As regards the optic lobe, *bantam* is involved in the proliferation of the OPC (Li and Padgett, 2012) where neuroepithelial cells differentiate into neuroblasts in a process in which both *miR-7* (Caygill and Brand, 2017) and *miR-8* (Morante et al., 2013) play an important role, while proper expression of *miR-210* was shown to be crucial for the correct neurogenesis and arborization of the l-LNvs in the optic lobe (Cusumano et al., 2018) (Figure 2B). In addition, several of these miRNAs (*bantam*, *miR-210*, *miR-263a/b*, *miR-279/996*) are involved in the regulation of circadian rhythms, further reinforcing the link between the proper functioning of the visual system and physiological circadian rhythmicity. Interestingly, almost all the discussed miRNAs have mammalian homologs and several of them show a highly or even perfectly conserved sequence in *Homo sapiens*, often sharing similar target genes and also showing similar physiological and pathological effects. The *miR-210* seed sequence is 100% identical between flies and humans (Weigelt et al., 2019) and numerous studies have suggested a potential role of *miR-210* in eye diseases, since it was found to be elevated in primary open-angle glaucoma patients (Liu et al., 2019) and it is linked to corneal epithelial repair (Kaplan et al., 2022). *miR-7* is perfectly conserved from annelids to humans and is highly expressed in the human brain. It participates in the development of the visual system regulating the expression of PAX6 which is an important mediator of ocular formation (Zhao et al., 2020). *miR-9* is one of the most highly expressed miRNAs in the vertebrate brain and it is involved in neurogenesis, balancing the proliferation of embryonic neural progenitor cells, also at the level of the retina (Coolen et al., 2013). The *miR-200* family, which constitutes the vertebrate homolog of *miR-8*, has emerged as an important regulator of neurogenesis and gliogenesis (Trümbach and Prakash, 2015) similarly to the role of *miR-8* in the *Drosophila* optic lobes. The *miR-183* family, which is the vertebrate homolog of *miR-263a* and *miR-263b*, is particularly expressed in the ciliated sensory organs (in mice it is expressed in the bipolar, amacrine, and photoreceptors cells), thus highlighting a conserved main role among different species (Pierce et al., 2008). In particular, the KO of these miRNAs results in retinal degeneration in mice (Lumayag et al., 2013). In conclusion, the architectural similarities between *D. melanogaster* and vertebrates’ visual system (Sanes and Zipursky, 2010) and the evolutionary conservation of the miRNAs involved in its development and modulation suggest the importance of the fruit fly as a model to further characterize the molecular and neuronal circuits in which miRNAs are involved in the specification and functioning of the visual system of higher organisms including humans. This in turn may pave the way for the identification of promising therapeutic targets for retinal and neurodegenerative diseases.
